# Human DNA polymerase α in binary complex with a DNA:DNA template-primer

**DOI:** 10.1038/srep23784

**Published:** 2016-04-01

**Authors:** Javier Coloma, Robert E. Johnson, Louise Prakash, Satya Prakash, Aneel K. Aggarwal

**Affiliations:** 1 Department of Structural & Chemical Biology, Mount Sinai School of Medicine, Box 1677, 1425 Madison Avenue, New York, NY 10029, USA; 2 Department of Biochemistry and Molecular Biology, 301 University Boulevard, University of Texas Medical Branch, Galveston, TX 77755-1061, USA

## Abstract

The Polα/primase complex assembles the short RNA-DNA fragments for priming of lagging and leading strand DNA replication in eukaryotes. As such, the Polα polymerase subunit encounters two types of substrates during primer synthesis: an RNA:DNA helix and a DNA:DNA helix. The engagement of the polymerase subunit with the DNA:DNA helix has been suggested as the of basis for primer termination in eukaryotes. However, there is no structural information on how the Polα polymerase subunit actually engages with a DNA:DNA helix during primer synthesis. We present here the first crystal structure of human Polα polymerase subunit in complex with a DNA:DNA helix. Unexpectedly, we find that portion of the DNA:DNA helix in contact with the polymerase is not in a B-form but in a hybrid A-B form. Almost all of the contacts observed previously with an RNA primer are preserved with a DNA primer – with the same set of polymerase residues tracking the sugar-phosphate backbone of the DNA or RNA primer. Thus, rather than loss of specific contacts, the free energy cost of distorting DNA from B- to hybrid A-B form may augur the termination of primer synthesis in eukaryotes.

DNA replication in eukaryotes requires the coordinated activity of three multi-subunit DNA polymerases (Pols): Polα, Polδ and Polε^1^,^2^,^3^,^4^. Polα/primase is a four-subunit enzyme (p180, p70, p49 and p58 in humans) whose activity is absolutely required for cell viability^5^,^6^,^7^,^8^. This complex assembles short RNA-DNA primers which are subsequently extended by the processive Polδ and Polε. The two primase (p49 and p58) subunits synthesize *de novo* a 7–12 ribonucleotides oligomer, the 3′ end of which is then intramolecularly handed-off to the Polα active site located in the large catalytic subunit (p180)^6^,^8^. The RNA primer is then extended using deoxyribonucleotides to form a strand of approximately 30 nucleotides (nts) in length (Fig. 1). As such, the Polα catalytic subunit encounters two types of DNA substrates during the primer synthesis: an RNA:DNA helix that is handed over to it by the primase subunits and then a DNA:DNA helix during subsequent DNA synthesis. Crystal structures of the catalytic cores of yeast and human Polα bound to RNA:DNA helices have been reported recently^9^,^10^. It has been suggested that Polα is specifically adapted to recognize the A-form RNA:DNA helix and that subsequent synthesis and encounter with B-form DNA leads to the termination of primer synthesis^9^. We present here for the first time a structure of the catalytic core of human Polα (hPolα) bound to a DNA:DNA helix.

Pols α, δ, and ε belong to the B-family of DNA polymerases^3^,^11^. Crystal structures of the catalytic subunits of Polα, Polδ, and Polε reveal a characteristic B-family polymerase fold comprised of a palm domain that carries the catalytic residues for dNTP addition, a fingers domain that drapes over the nascent base pair, a thumb domain that makes contacts in the DNA minor groove, and an N-terminal domain (NTD)^9^,^10^,^12^,^13^,^14^. The catalytic cores of Polδ and Polε also contain an active exonuclease domain, whereas in Polα the exonuclease domain is inactive due to a lack of catalytic residues. We show here how Polα copes with a DNA:DNA helix during primer synthesis. Surprisingly, the DNA:DNA helix in contact with the polymerase adopts a mixed A-B conformation that has implications for the termination of primer synthesis.

## Results

### Structure determination

**Figure 2:**
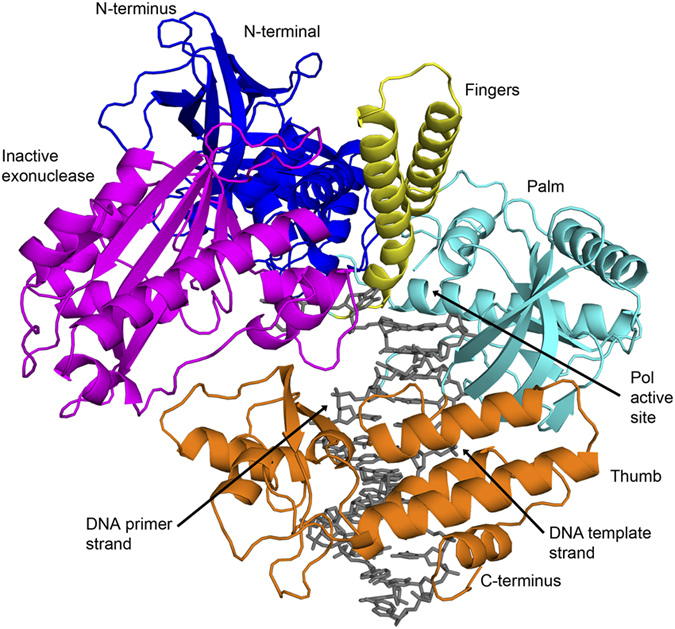
Structure of hPolα binary complex. The hPolα palm, fingers, thumb, exonuclease and N-terminal domains are shown in cyan, yellow, orange, magenta and blue respectively. DNA is shown in grey color. The polymerase (Pol) active site is labeled.

The catalytic core of hPolα (residues 339 to 1259 of the p180 subunit) was crystallized with a 13-nt primer DNA/18-nt template DNA from a mix containing Mg^2+^ and dCTP. The cocrystals diffract to 3.3 Å resolution with synchrotron radiation (Brookhaven National Laboratory) and belong to space group P2_1_ with unit cell dimensions of a = 137.04 Å, b = 132.03 Å, c = 163.14 Å, and β = 109.13° (Table 1). There are four polymerase-DNA (binary) complexes in the crystallographic asymmetric unit. The structure was solved by molecular replacement (MR) using the yeast homolog of p180 (PDB code 4FYD) as a search model^9^. Although the primer/template and dNTP were omitted from the MR search model, there was clear electron density for the primer and template DNA strands in the initial electron density maps derived from the MR phases. However, electron density for the fingers domain (residues 908–965) was relatively weak. To obtain unbiased maps for this region, the first few rounds of refinement and model building were performed by omitting the fingers domain. Unlike the ternary (polymerase-RNA/DNA-incoming nucleotide) structures where the fingers domain is draped over the nascent base-pair, the fingers domain in our structure is in the open conformation (Fig. 2). Our complex may have crystallized in the binary state because of crystals contacts, whereby several residues (Asn935, Arg945 and Tyr942) on the fingers domain are involved in contacts with neighboring molecules, favoring the fingers domain in the open conformation. The final refined model (R_free_ of 22.9%; R_work_ of 18.3%) contains four protein chains, eight DNA strands, and 100 solvent molecules. The structure has good stereochemistry (Table 1 and Fig. S1), with 96% of the residues in the most favored regions of the Ramachandran plot. The four binary complexes in the asymmetric unit are very similar in structure, with the polymerases superimposing with an average rmsd of ~0.9 Å (for 857 Cαs). We describe below the structure corresponding to polymerase chain A bound to DNA chains B (primer) and C (template).

### Overall arrangement

The Polα catalytic core surrounds the DNA primer/DNA template (Fig. 2). The palm domain (residues 809–881, 964–1076) interacts with the replicative end of the primer-template and carries the active site residues (Asp860 and Asp1004) for DNA synthesis. The fingers domain (residues 909–963) is composed of two long α-helices that hover above the templating base. The thumb domain (residues 1077–1245) is composed of two subdomains that grip the duplex portion of the primer-template, making extensive contacts with the DNA through the minor groove (Fig. 2). The inactive exonuclease domain lies on the opposite side of the DNA as the thumb domain, extending towards the major groove (Fig. 2). The catalytic residues associated with exonuclease activity in other B-family polymerases (Asp321, Glu323 and Asp407 in Polδ, for example^12^) are absent, lending to the lower fidelity Polα when compared to Polδ or Polε^15^. The NTD bridges the exonuclease and fingers domains. The NTD has been suggested to bind RNA and DNA in Polδ^12^, but in Polα its role is unclear. There are no magnesium ions in the active site despite their inclusion in the crystallization mix. This reflects the absence of incoming nucleotide in the active site, the triphosphate moiety of which (along with active side residues) is typically associated with magnesium ions in the active site of polymerase ternary structures^16^.

**Figure 3:**
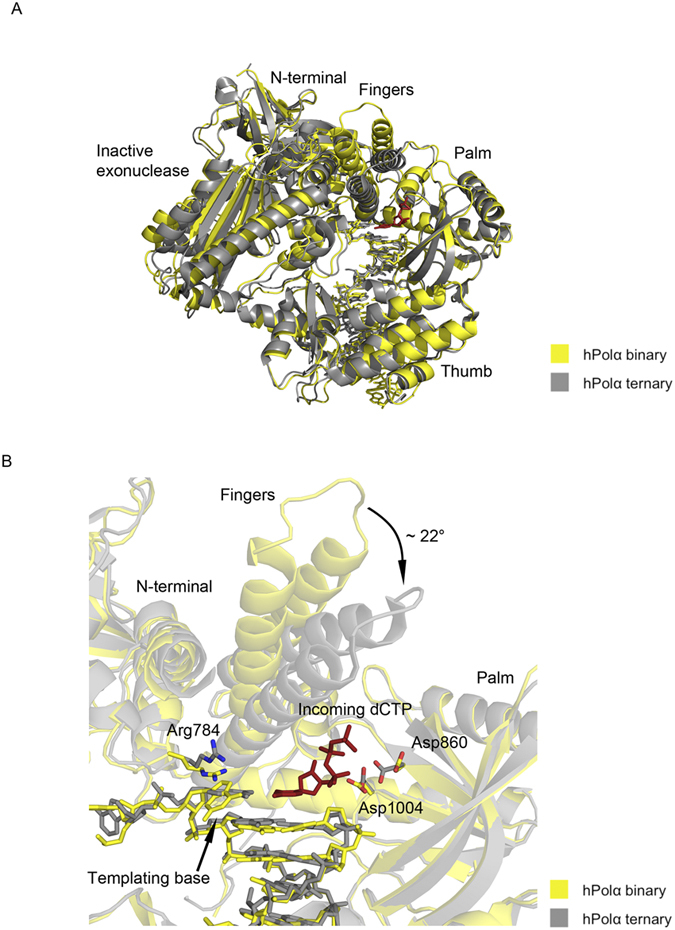
Comparison between binary and ternary structures of hPolα. (**A**) The hPolα binary complex bound to DNA:DNA is colored yellow and the ternary complex bound to RNA:DNA (PDB code 4 QCL) is colored gray. The palm, fingers, thumb, exonuclease and N-terminal domains are labeled. (**B**) Close-up view of the hPolα active site. Side chains for residues Arg784, Asp860 and Asp1004 are shown with oxygen atoms in red and nitrogen atoms in blue. The incoming nucleotide (dCTP) from the ternary structure is shown in red.

When compared to the human Polα ternary structure, the fingers domain in our binary structure is rotated outwards by ~22° (Fig. 3A). The open (binary) to closed (ternary) transition in the fingers domain is a hallmark of replicative DNA polymerases^17^, reflecting the favorable interactions established between residues on the fingers domain and the triphosphate moiety of incoming dNTP. The configuration of the other domains is generally similar to that in the yeast and human Polα ternary structures^9^,^10^. For example, a superposition based on the palm domain in our binary structure and that in the human ternary structure lends to rmsds of 0.72 Å, 3.53 Å and 1.12 Å between the thumb domains, the exonuclease domains, and the NTDs, respectively. Thus, even though the binary and ternary structures were determined with different nucleic acid substrates, the mode of binding is unexpectedly similar.

Intriguingly, the templating guanine base is rotated about its glycosidic bond to the *syn* conformation. It is stabilized in this conformation by stacking interactions with the side chain of Arg784 from the NTD (Fig. 3B). By contrast, in the Polα ternary structures^9^,^10^, the templating guanine adopts the *anti* conformation for Watson-Crick base-pairing with incoming dNTP. Of the active site residues, Asp1004 is oriented in the same fashion as in the ternary structure, while Asp860 is rotated away from the active site. Taken together, the opening and closing of the fingers domain is the dominant conformational transition between Polα binary and ternary complexes, with local conformational changes restricted primarily to the templating base and the active site residues.

### DNA conformation

**Figure 4:**
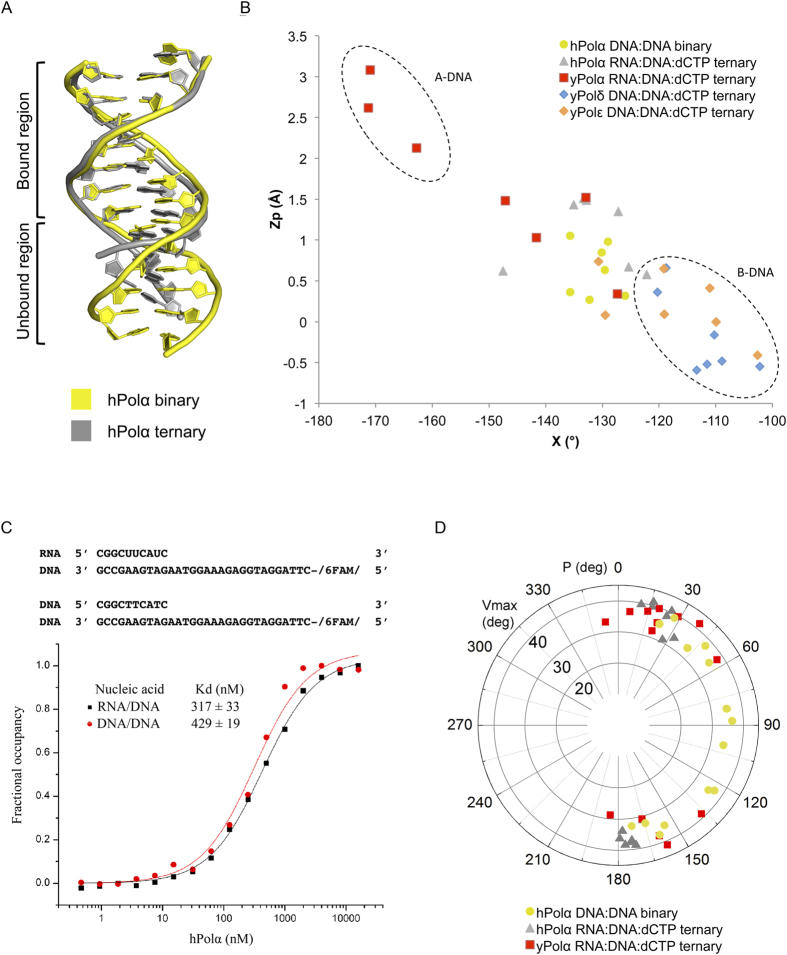
Conformation of nucleic acid. (**A**) Comparison of the DNA conformations of the hPolα binary complex (yellow) and the hPolα ternary complex (gray). The bound and unbound regions are marked in the figure. (**B**) Scatter plot of Z_p_, the mean z-coordinate of the backbone phosphorous atoms with respect to individual dinucleotide reference frames, against the mean value for the four χ torsion angles at each dinucleotide step. The values for 7 DNA:DNA base steps bound to hPolα in the binary complex are shown as yellow circles. The 7 RNA:DNA base steps bound to hPolα in the ternary complex (PDB code 4 QCL) are shown as gray triangles. The values for RNA:DNA steps bound to yPolα (red squares), DNA:DNA steps bound to yPolδ (blue diamonds) and DNA:DNA steps bound to yPolε (orange diamonds) are also plotted. (**C**) Binding affinities of hPolα for DNA/DNA and RNA/DNA measured by a fluorescence anisotropy assay. The fraction of DNA/DNA or RNA/DNA bound is plotted versus hPolα concentration (logarithmic scale) in order to determine the dissociation constants. (**D**) Distribution of the pseudorotational phase angle P and puckering amplitude v_max_ of the base steps in contact with hPolα in the binary complex (yellow circles), with hPolα in the ternary complex (gray triangles) and with yPolα in the ternary complex (red squares).

Strikingly, the portion of DNA:DNA helix in contact with the polymerase has a mixed A-B conformation while the portion of the duplex outside of the contact region remains in the standard B-form (Fig. 4A and S2). A-form DNA is wider and shorter than B-form DNA, with a diameter of 23 Å versus 20 Å and a rise per base of 2.56 Å versus 3.38 Å, respectively^18^. Also, the base pairs in A-form DNA are more inclined and displaced form the helix axis. The pathway between the two DNA forms is most easily visualized as a plot of torsion angles δ and χ or as a plot of phosphate displacement (Z_p_) and χ^9^,^19^,^20^,^21^. From Fig. 4B, the Polα catalytic core imposes a mixed A-B conformation on the DNA:DNA helix in contact with it, close to the conformation observed when yeast or human Polα binds an RNA:DNA helix. One difference is that in the latter the RNA:DNA helix outside the contact region is A-form, reflecting the tendency of isolated RNA:DNA helices to adopt A-form^9^,^10^. The preference of Polα for A-B-like DNA conformation is in marked contrast to Polδ and Polε, wherein the DNA duplex in contact with the polymerases remains predominantly B-form^12^,^13^,^14^.

To further test the idea that Polα prefers a similar DNA conformation when it binds a RNA:DNA or a DNA:DNA helix, we measured the binding constants (K_d_s) for both substrates. The K_d_s were measured by fluorescence anisotropy using a fluorescein labeled 10-nt RNA-primer/30-nt DNA-template and a 10-nt DNA primer/30-nt DNA-template. As shown in Fig. 4C, the Polα catalytic core binds the two substrates with similar affinities, namely with a K_d_ of ~320 nM to the RNA:DNA helix and with a K_d_ of ~430 nM to the DNA:DNA helix.

The A- and B-conformations are typically associated with a C3′ endo and C2′ endo sugar puckers, respectively^18^. The range of sugar conformations in RNA or DNA is best viewed as a circular plot of the pseudorotation angle P (0 to 360°) and the maximum out of plane deviation V_max_ 18,22. From Fig. 4D, sugar conformations in the Polα RNA:DNA ternary structures are concentrated around P values of ~30° and ~150° in the DNA and RNA strands, respectively. Interestingly, the sugars in our DNA:DNA binary structure span a range of P values between ~30° and ~150° in accord with a mixed A-B-like conformation.

### Protein-DNA Interactions

**Figure 5:**
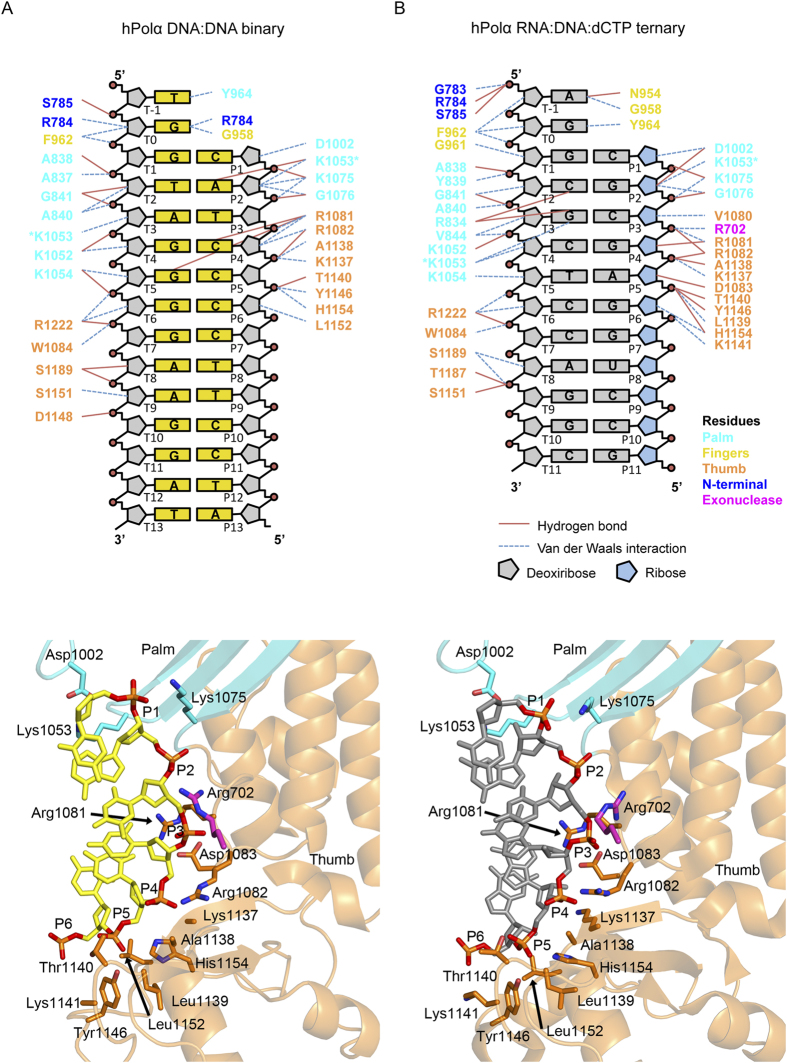
Protein-nucleic acid interactions. (**A**) Top, a schematic drawing of protein-DNA interactions in the hPolα binary complex. Residues from the palm are colored in cyan, from the thumb in orange, from the exonuclease in magenta, from the N-terminal in blue, and from the fingers in yellow. Only direct contacts between the protein and the nucleic acid are shown. Hydrogen bonds are indicated with blue lines (determined by interaction distances <3.2 Å), and van der Waals interactions are indicated with brown lines (determined by interaction distance <4.2 Å). The residues marked with an asterisk are represented more than once in the diagram. Templates bases are labeled T-1 to T13 and primer bases P1 to P13. Bottom, a view of the DNA primer interacting with residues from the palm and thumb domains. (**B**) Top, a schematic drawing of protein-DNA and protein-RNA interactions in the hPolα ternary complex with incoming dCTP (PDB code 4 QCL). The deoxyriboses are colored gray and the riboses are colored blue. Bottom, a view of the RNA primer interacting with residues from the palm and thumb domains.

The palm and thumb and domains interact extensively with the duplex portion of the template-primer. For convenience, we refer to the terminal base pair at the 3′ end of the primer as T_1_-P_1_, and subsequent base pairs in the duplex as T_N_-P_N_ (where T and P refer to template and primer strands, respectively, and the subscript N refer to the number of base pairs from the templating base position). The contacts are limited to the sugar-phosphate backbone of the DNA duplex and occur primarily through the minor groove (Fig. 2). A key question is how contacts to the DNA primer compare to those observed with an RNA primer in previous Polα structures? Surprisingly, the contacts are remarkably similar (Fig. 5). For example, the same (or analogous) residues track the sugar-phosphate backbone of the DNA or RNA primer strand (Fig. 5). These “tracking” residues lie at the nexus between the palm and thumb domains, and include a pair of invariant arginines (Arg1081, Arg1082) that bracket the phosphate group of nucleotide P_4_. In addition, residues near the tip of the thumb domain (Lys1137, Ala1138, Thr1140, Tyr1146, His1154 and Leu1152) interact with the more downstream portion of the primer, namely nucleotides P_4_-P_6_. Interactions with the template strand are also very similar, including contacts from three consecutive lysines (Lys1052, Lys1053 and Lys1054) from the palm domain and several residues from the thumb domain (Trp1084, Asp1148, Ser 1151, Ser1189, and Arg1222) (Fig. 5). The unpaired segment of the template strand lies between the base of the fingers and the exonuclease domain – oriented away from the DNA duplex. Altogether, the polymerase interacts with nucleotides P_1_-P_6_ of the primer strand and T_1_-T_10_ of the template strand that, as noted previously^9^, is a smaller footprint than that observed with catalytic subunit of Polδ.

## Discussion

The Polα/primase complex is an essential component of eukaryotic DNA replication - required for the initiation of Okazaki fragment synthesis. The primase subunits synthesize a 7–12 RNA oligomer that is then intra-molecularly handed-off to the polymerase subunit for DNA synthesis - leading to a composite RNA-DNA primer of ~30 nt in length. A key question is how synthesis of this primer is terminated? Since the Polα polymerase subunit lacks the proofreading exonuclease function of Polδ and Polε it would be advantageous to limit the extent of DNA synthesis on the primer. Interestingly, DNA synthesis itself has been suggested as a basis for the termination of primer synthesis^9^. The Polα catalytic subunit is postulated to specifically recognize the A-form RNA:DNA helix and the ensuing synthesis of B-form DNA:DNA helix is suggested to disfavor the continuous engagement of the polymerase with the primer^9^. There is no structural information, however, as to how Polα copes with a DNA:DNA helix during primer synthesis.

We present here the first crystal structure of human Polα polymerase subunit in complex with a DNA:DNA helix. The structure sheds new light into how Polα engages a DNA:DNA helix during primer synthesis. Surprisingly, the portion of the DNA:DNA helix in contact with the polymerase is not in a B-form but in a hybrid A-B form. By contrast, the portion of the DNA:DNA helix outside the contact region remains in standard B-form. This suggests a model where Polα imposes a hybrid A-B-like conformation on the DNA:DNA helix rather than any major rearrangement in the polymerase to fit a B-form DNA. Almost all of the contacts noted previously with an RNA primer are preserved with a DNA primer^9^,^10^. The same set of polymerase residues track the sugar-phosphate backbone of the DNA or RNA primer, making nearly identical polar and non-polar interactions. Compared to Polδ, the nucleic acid binding cavity in Polα is significantly wider and better able to accommodate hybrid A-B form DNA. For example, measured as the distance between Cαs of Lys1052 and Asp1148 of human Polα and the homologous residues Lys813 and Asn899 yeast Polδ, the width is ~31 Å in Polα and ~26 Å in Polδ (Fig. S3). Part of this increase in width in Polα is due to a ~15–20° rotation in the second subdomain of the thumb domain.

Altogether, there does not appear to be a significant loss of contacts when Polα engages a DNA:DNA helix versus a RNA:RNA helix, as also reflected in similar binding affinities for a DNA:DNA or an RNA:DNA helix (Fig. 4C). Nonetheless, it is possible that the free energy cost of distorting a DNA:DNA helix from B- to A-B conformation increases as synthesis continues from the RNA primer. Because of strong communication between neighboring segments of nucleic acids via stacking and other interactions, the conformation of one segment will influence the other. For example, it has been shown that inclusion of a single ribonucleotide at the 3′ end of a short DNA segment is sufficient to drive the DNA from B- to A-form^23^. As DNA synthesis continues from an RNA primer, the gradual accumulation of B-DNA may make it progressively harder for the DNA region in contact with Polα to acquire the A-B form. Whether this is the case will require more detailed kinetic and binding studies with different lengths of RNA:DNA primers.

In conclusion, we show here that a DNA:DNA helix transitions from B- to mixed A-B form when it engages the Polα catalytic subunit. Since the conformation of DNA can be sensitive to a number of features^18^,^24^, including sequence, epigenetic modifications, ions, and local water activity, it will be interesting to see how these different features impact primer extension during DNA replication.

## Methods

### Protein and DNA preparation

The catalytic core of human p180 (residues 338–1259) harboring a N-terminal hexahistidine SUMO (His_6_-SMT) tag was expressed in the *E. coli* BL21 (DE3) Star pLysS strain. Protein was purified by affinity chromatography with His60 Ni Superflow Cartridge. The His_6_-SMT was removed via overnight incubation with SUMO protease, and the protein was subsequently purified via ion-exchange (HiTrap Q column) and size-exclusion (Superdex 200) chromatography. Purified protein was concentrated and stored at −80 °C until further use.

The primer and template strands used for crystallization were purified by anion exchange on a MonoQ column, desalted and lyophilized before crystallization. Purified 13-nt primer harboring a dideoxycytosine at the 3′ end (ATCCTTCCCCTACdd) was mixed with purified 18-nt template (TAATGGTAGGGGAAGGAT) in 1:1 ratio and annealed to yield a 13/18 template-primer duplex DNA with one replicative end.

### Cocrystallization

The p180 (core) ternary complex was prepared by mixing purified hPolα (388–1259) and the 13/18 template – primer DNA duplex in the ratio of 1:1, followed by the addition of dCTP and MgCl_2_ to final concentrations of 5 and 10 mM respectively. Crystals were obtained in a solution containing 10% polyethylene glycol 4000, 0.2 M sodium citrate, 10% isopropanol, 0.1 M sodium citrate buffer (pH = 5.5). For data collection, crystals were cryoprotected by gradually replacing the mother liquor in the drop for a new one containing 20% glycerol and then flash frozen in liquid nitrogen. X-ray data on cryocooled crystals were measured at the National Synchrotron Light Source (NSLS, beamline X25) of Brookhaven National Laboratory at a wavelength of 1.1 Å. Data sets were indexed and integrated using iMOSFLM^25^. Crystals diffract to 3.3 Å and belong to space group P1 21 1 with unit cell dimensions of a = 137.04 Å, b = 132.03 Å, c = 163.14 Å and α = 90° β = 109.13°, γ = 90°. Matthew’s coefficient suggested four protein-DNA molecules in the asymmetric unit (based on 59% solvent by volume).

### Structure determination and refinement

The structure of p180 was solved by molecular replacement (MR), using the program Mr. Bump^26^. The software was able to find a solution using as a search model the structure of the ternary complex of the yeast homolog^9^. The first round of rigid body refinement and map calculation was carried out without the template/primer/dNTP using the program PHENIX^27^. The electron density maps (2Fo-Fc and Fo-Fc) showed unambiguous densities for the template and primer DNA strands, which were then included in the model for subsequent refinement. Standard individual *xyz* refinement was used with individual B factors along with TLS refinement (32 groups). During the first rounds of refinement torsion-angle non-crystallographic symmetry (NCS) restraints were used. The NCS restraints were removed during the later stages of refinement to avoid missing local differences in the 4 molecules of the asymmetric unit. Weight optimization of the geometry and B-factor restraints were carried out towards the end of refinement to prevent over fitting. The iterative rounds of refinement and water picking were performed with PHENIX and model building with program Coot^28^. The final refined model (R_free_ of 22.9%; R_work_ of 18.3%) contains four protein chains (A, residues 338–673, 680–808, 836–881, 898–1245; D, residues 338–674, 679–807, 837–881, 896–1255; G, residues 338–675, 678–808, 838–881, 895–1244; J, residues 338–673, 679–808, 836–881, 897–1245), eight DNA strands (B (template), residues 3–18; C (primer), 1–13; E (template), residues 2–18; F (primer), 1–13; H (template), residues 2–15; I (primer), 4–13; K (template), residues 3–18; L (primer), 1–13), and 100 solvent molecules. The model has good stereochemistry as shown by MolProbity^29^ with >95.6% of all residues in allowed regions of the Ramachandran plot and less than 0.1% in the disallowed regions. Coordinates have been deposited in the Protein Data Bank with the accession code 5IUD. Figures were prepared using PyMol (The PyMOL Molecular Graphics System, Version 1.7.4.4, Schrödinger, LLC).

### Structural analysis

Protein structures were aligned and superimposed using MUSTANG and SuperPose Version 1.0^30^,^31^. Web 3DNA (w3dna.rutgers.edu) was used for analysis of DNA helical parameters^32^.

### Fluorescence anisotropy

6-carboxyfluorescein (6-FAM)-labeled template (5′-CTTAGGATGGAGAAAGGTAAGATGAAGCCG-3′) and primer, RNA (5′-CGGCUUCAUC-3′) or DNA (5′-CGGCTTCATC-3′) oligonucleotides were purchased PAGE purified from IDT Technologies (Coralville, IA). Purified labeled 30 mer oligonucleotide was mixed with 10 mer RNA or DNA oligonucleotide in 1:1 ratio and annealed by heating to 95 °C and permitting the sample to cool to room temperature to yield a 10-nt RNA-primer/30-nt DNA-template or 10-nt DNA-primer/30-nt DNA-template with one replicative end. Fluorescence emission intensities were collected on a Panvera Beacon 2000 fluorescence polarization system (at 23 °C), and the anisotropy values calculated as previously described^33^. Each reaction sample (total volume of 200 μl) consisted of 2 nM of 5′ FL-labeled RNA/DNA or DNA/DNA and increasing concentrations of the protein (from 0.1 nM to 16000 nM) in a binding buffer containing 50 mM Bis-Tris (pH 6.5), 100 mM NaCl and 5 mM MgCl_2_. The samples were left to equilibrate at room temperature for >30 min before the fluorescence anisotropy values were measured. Anisotropy values were referenced against a blank buffer at the beginning of each experiment to account for background correction. Anisotropy values were normalized by first subtracting the anisotropy value with no protein added and then dividing by the maximum anisotropy value for a particular RNA/DNA or DNA/DNA series. Fractional occupancy values were then plotted versus protein concentration, and the data fitted by nonlinear least-squares regression, by using Origin 7 (OriginLab), to the following equation:


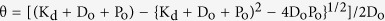


where θ is the fraction of nucleic acid bound, D_o_ is the total concentration of nucleic acid, P_o_ is the total protein concentration, and K_d_ is the dissociation constant.

## Additional Information

**How to cite this article**: Coloma, J. *et al.* Human DNA polymerase a in binary complex with a DNA:DNA template-primer. *Sci. Rep.* **6**, 23784; doi: 10.1038/srep23784 (2016).

## Supplementary Material

Supplementary Information

## Figures and Tables

**Figure 1:**
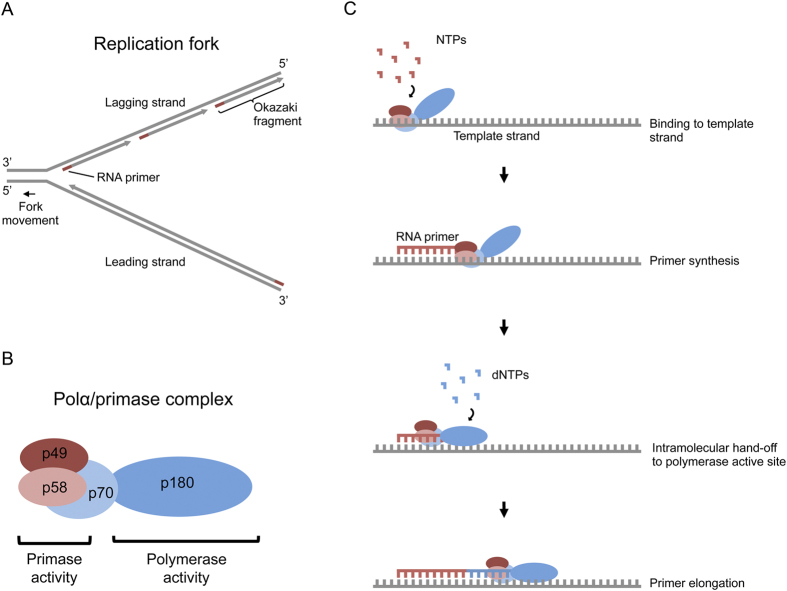
Polα/primase mediated assembly of RNA-DNA primers during DNA replication. (**A**) Because DNA synthesis proceeds in the 5′ to 3, direction, the replication fork is asymmetrical, with continuous DNA synthesis on the leading strand and discontinuous DNA synthesis (via Okazaki fragments) on the lagging strand. (**B**) The Polα/primase complex is composed of four subunits (p180, p70, p49 and p58 in humans). (**C**) The Polα/primase complex assembles RNA-DNA primers required to initiate DNA synthesis on leading and lagging strands in eukaryotes.

**Table 1:** Data collection and refinement statistics.

	Human Polymerase Alpha binary complex
Data collection	
Space group	P 1 21 1
Cell dimensions	
a, b, c (Å)	137.04, 132.03, 163.14
α, β, γ (°)	90, 109.13, 90
Resolution (Å)	60.6–3.3 (3.42–3.3)
R_merge_	0.053 (0.365)
I/σI	8.14 (1.93)
Completeness (%)	99.6 (99.4)
Redundancy	1.9 (1.9)
Refinement	
Resolution (Å)	3.3
No. reflections	82313 (8127)
R_work_/R_free_	0.183 (0.257)/0.229 (0.318)
No. atoms	28203
Macromolecules	28100
Water	100
B-factors	108.98
Macromolecules	109.10
Water	75.96
R.m.s. deviations	
Bond lengths (Å)	0.002
Bond angles (°)	0.51

Statistics for the highest-resolution shell are shown in parentheses.
