# Supplementary material for: Human DNA polymerase α in binary complex with a DNA:DNA template-primer
Source: Sci Rep. 2016 Apr 1;6:23784. doi: 10.1038/srep23784 (PMC4817131; doi:10.1038/srep23784)
Supplement: Supplementary Information [file srep23784-s1.pdf]

Human DNA polymerase α in binary complex with a DNA:DNA template-primer

Supplementary information

Javier Coloma[1], Robert E. Johnson[2], Louise Prakash[2], Satya Prakash[2], Aneel K. Aggarwal[1].

[1]Department of Structural & Chemical Biology
Mount Sinai School of Medicine
Box 1677
1425 Madison Avenue
New York, NY 10029, USA.

[2]Department of Biochemistry and Molecular Biology
301 University Boulevard
University of Texas Medical Branch
Galveston, TX 77755-1061, USA

[*] Correspondence: Aneel.Aggarwal@mssm.edu

Supplementary figure 1

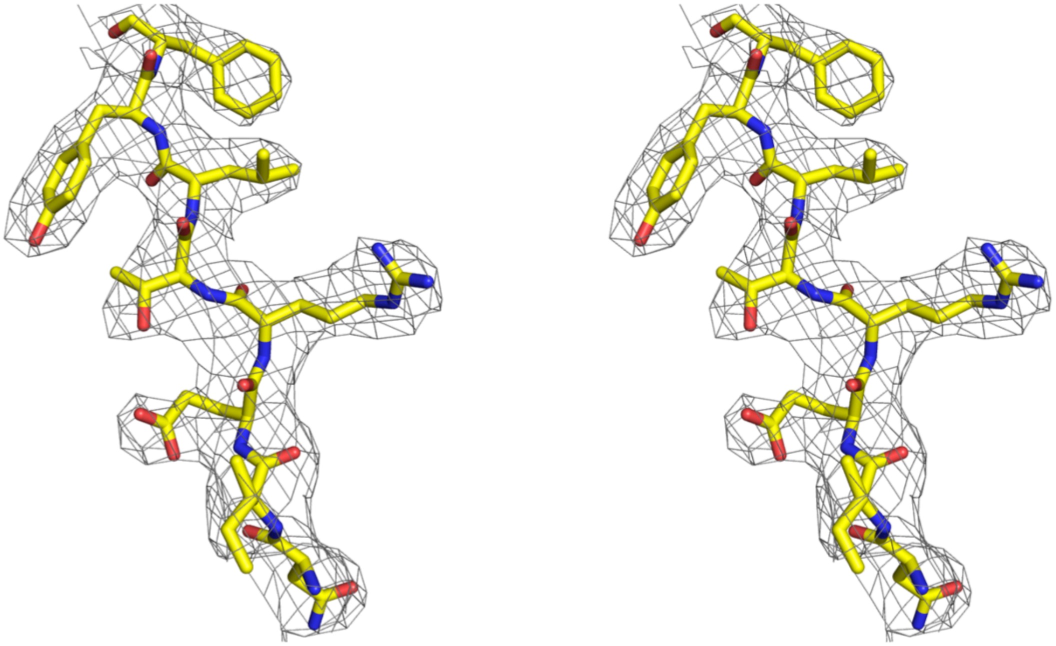

Figure S1. Stereo image of hPolα residues 382 to 389. The 2Fo−Fc electron density map at 3.3Å resolution is displayed at contour level of 1.0σ. The map was generated by Phenix and displayed using Coot. Protein is drawn as sticks and the electron density maps are drawn as mesh.

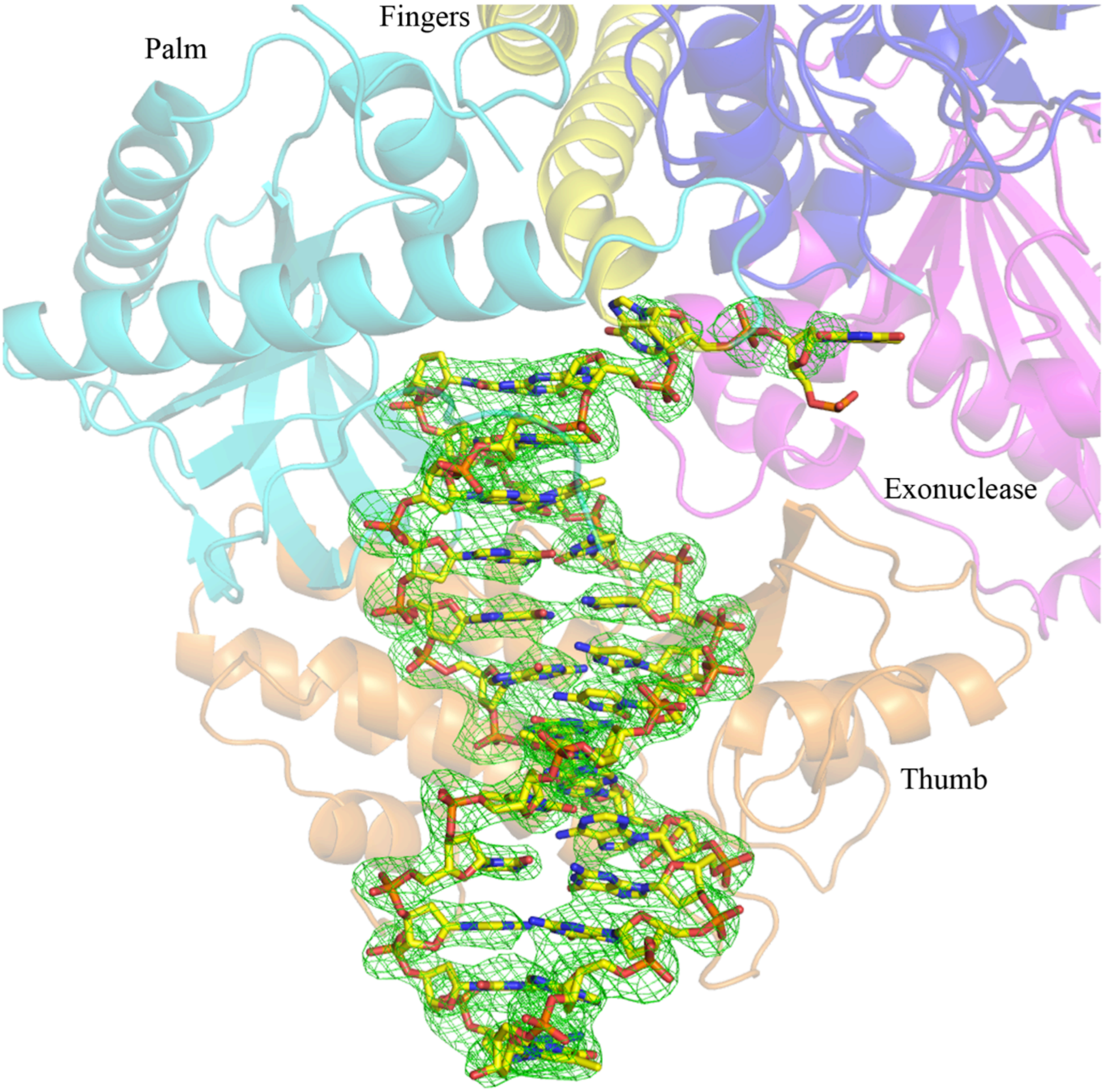

Figure S2. Simulated annealing Fo-Fc omit map. The omit map (contoured at 3σ) was generated with the DNA atoms (chains B and C) omitted and is represented as a green mesh. The hPolα palm, fingers, thumb, exonuclease and N-terminal domains are shown in cyan, yellow, orange, magenta and blue respectively. DNA is shown in elemental colors. There was no density for the first three nucleotides of the template strand; only nucleotides 4-18 were modeled.

Supplementary figure 3

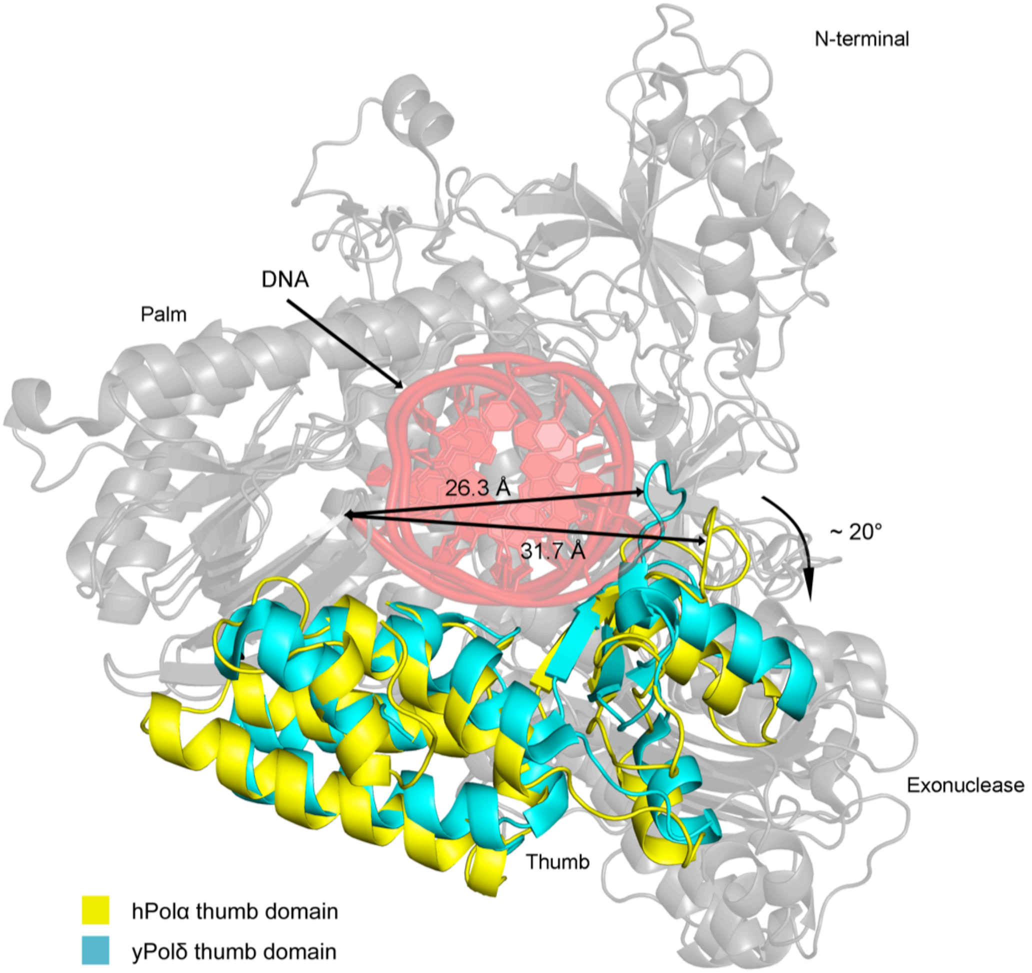

Figure S3. Comparison of DNA binding cavity in hPolα and yPolδ. The hPolα binary and yPolδ ternary structures were superposed to compare their DNA binding cavities. All proteins domains except the thumbs are shown in gray. The DNA base pairs in contact with the proteins are shown (red). The hPolα thumb domain is colored yellow and the yPolδ thumb domain is colored cyan. The distances between Lys1051 and Asp1148 in hPolα and the homologous amino acids in yPolδ, Lys812 and Asn899 are marked with black arrows. Palm, N-terminal, endonuclease and thumb domains are labeled.
